# The association between DNA methylation of *6p21.33* and *AHRR* in blood and coronary heart disease in Chinese population

**DOI:** 10.1186/s12872-022-02766-8

**Published:** 2022-08-13

**Authors:** Liya Zhu, Chao Zhu, Jinxin Wang, Rongxi Yang, Xiaojing Zhao

**Affiliations:** 1 Department of Epidemiology and Biostatistics, School of Public Health, Nanjing Medical University, Nanjing, 211166 China; 2 Department of Cardiology, Beijing Friendship Hospital, Capital Medical University, 95 Yong’an Road, West District, Beijing, 100050 China; 3 Department of Cardiology, The Second Medical Centre, Chinese PLA General Hospital, Beijing, 100853 China; 4 Military Translational Medicine Lab, Medical Innovation Research Division, Chinese PLA General Hospital, Beijing, 100853 China; 5 Beijing Key Laboratory of Chronic Heart Failure Precision Medicine, Medical Innovation Research Division, Chinese PLA General Hospital, Beijing, 100853 China

**Keywords:** Coronary heart disease, *AHRR*, *6p21.33*, DNA methylation, Peripheral blood

## Abstract

**Background:**

Early detection could significantly improve the prognosis of coronary heart disease (CHD). In-invitro diagnostic technique may provide a solution when sufficient biomarkers could be identified. Pertinent associations between blood-based aberrant DNA methylation and smoking, the pathogenesis of atherosclerosis, and CHD have been robustly demonstrated and replicated, but that studies in Chinese populations are rare. The blood-based methylation of aryl-hydrocarbon receptor repressor (*AHRR*) cg05575921 and *6p21.33* cg06126421 has been associated with cardiovascular mortality in Caucasians. Here, we aim to investigate whether the *AHRR* and *6p21.33* methylation in the blood is associated with CHD in the Chinese population.

**Methods:**

In this case–control study, 180 CHD patients recruited at their first registration in our study center, and 184 controls randomly selected from the people who participated in the annual health examination were enrolled. Methylation intensities of 19 CpG sites, including *AHRR* cg05575921, *6p21.33* cg06126421, and their flanking CpG sites, were quantified by mass spectrometry. The association between methylation intensities and CHD was estimated by logistic regression analyses adjusted for covariant.

**Results:**

Compared to the controls, lower methylation of 6p21.33_CpG_4.5/cg06126421 was independently associated with increased odds of being a CHD patient (OR per − 10% methylation = 1.42 after adjustment for age, gender, and batch effect; *p* = 0.032 by multiple testing corrections). No association between blood-based *AHRR* methylation and CHD was found.

**Conclusions:**

*6p21.33* methylation exhibits a significant association with CHD. The combination of *6p21.33* methylation and conventional risk factors might be an intermediate step towards the early detection of CHD.

**Supplementary Information:**

The online version contains supplementary material available at 10.1186/s12872-022-02766-8.

## Introduction

Coronary heart disease (CHD) is a gene-environment interacted disease mainly caused by atherosclerosis which is usually asymptomatic in the early stage [1–3], producing immense health and economic burdens on a global scale [4, 5]. Hypertension, dyslipidemia, obesity, diabetes, and smoking, have been well-known as risk factors for CHD [6, 7]. A series of biomarkers for cardiovascular disease have been identified, such as high-sensitivity C-reactive protein (hsCRP) [8], lipoprotein-associated phospholipase A2 (Lp-PLA2) [9], myeloperoxidase (MPO) [10], B-type natriuretic peptide (BNP), N-terminal prohormone BNP (NT-pro BNP) [11], leukocyte counts [12], as well as plasma metabolomics and circulating micro-RNAs [13, 14]. These traditional biomarkers have their specific application value but cannot fulfill the needs of risk evaluation for CHD, which is a complex disease affected by genetic, epigenetic, environmental risk factors, and other factors. Therefore, it would be meaningful to identify new potential CHD-related markers which may improve the chance to detect CHD.

DNA methylation is the covalent methylation of cytosine C5 in CpG dinucleotide involved in inherent and acquired transcriptional inhibition of genes, which occur independently of the DNA sequence [15]. As a reversible epigenetic modification, DNA methylation is a dynamic process related to environmental exposure [16]. It also serves as an important cellular regulatory mechanism that regulates gene expression associated with inflammation and atherosclerosis [17, 18]. Previous researches have shown that aberrant DNA methylation in the leukocytes is related to inflammation and the pathogenesis of atherosclerosis, and subsequently causes increased mortality in cardiovascular diseases [17–20]. Additionally, aberrant methylation patterns in blood have also been reported in CHD by candidate gene approaches [21–23]. However, the clinical value of the identified biomarkers remains limited, and the association between the epigenetic landscape and CHD is not completely understood.

The protein encoded by the aryl-hydrocarbon receptor repressor (*AHRR*) gene participates in the aryl hydrocarbon receptor (AhR) signaling cascade, which mediates dioxin toxicity, and is involved in the regulation of cell proliferation and differentiation [24, 25]. Smoking can trigger the production of AhR, which mediates dioxin toxicity and other pathological effects [26, 27]. Two epigenome-wide studies by Infinium Human Methylation Illumina 450 K BeadChip have revealed the association between smoking and blood-based DNA methylation in *AHRR* (cg05575921) and *6p21.33* (cg06126421) in the European population [28, 29]. Smoking is a major preventable risk factor for atherosclerosis and cardiovascular diseases [30, 31]. Reynolds et al. [32] further disclosed the association between smoking-responsive methylation of *AHRR* in the monocytes and subclinical atherosclerosis in a multi-ethnic study with 1,256 participants in the United States. In 2016, a prospective cohort study in Germany reported a strong association between cardiovascular mortality and a score based on the methylation intensity of two CpGs (*AHRR* cg05575921 and *6p21.33* cg06126421) [20]. Follow-up studies also suggested *AHRR* methylation in blood as a biomarker for cardiovascular disease in the Caucasian population, such as myocardial infarction, ischemic heart disease, ischemic stroke, and heart failure, as well as a predictor of the risk of all-cause mortality [33–36]. So far, there is no report about the association between the methylation of *6p21.33* (cg06126421) and the risk of cardiovascular disease in Chinese populations.

Hereby, the associations between CHD and the methylation of *6p21.33* (cg06126421) and *AHRR* (cg05575921) were investigated by a case–control study in the Chinese population. The correlations between DNA methylation and lifestyles, and historical treatments were also examined.

## Methods

### Study population

This investigation is based on a case–control study, details of which have been reported elsewhere [37]. Briefly, 180 patients with CHD and 184 controls were collected from the Chinese PLA General Hospital from 2018 to 2019. All the CHD patients were recruited at their first registration in our study center. Their histories of medical treatment were also recorded. Controls were recruited from people who participated in the annual health examination. Baseline characteristics for CHD cases and controls were listed in Table 1.

**Table 1:** Comparison of baseline characteristics between CHD cases and controls

Characteristics	All (N = 364)	Groups		*p-*value^a^
		Controls (N = 184)	CHD cases (N = 180)	
Age (median, IQR)	64(58–70)	63(57–68)	66(58–73)	**0.008**
Male (%)	223(61.3%)	114(62.0%)	109(60.6%)	0.784
Hypertension (%)	214(58.8%)	84(45.7%)	130(72.2%)	**2.00E**−**06**
Diabetes (%)	107(29.4%)	45(24.5%)	62(34.4%)	0.063
Smoking (%)	126(34.6%)	53(28.8%)	73(40.6%)	**0.027**
Drinking (%)	118(32.4%)	66(35.9%)	52(28.9%)	0.116
TC, mmol/L	4.01(3.38–4.8)	4.26(3.61–5.06)	3.81(3.25–4.42)	**0.001**
TG, mmol/L	1.35(1.00–1.95)	1.39(1.06–2.17)	1.30(0.95–1.86)	0.147
HDL, mmol/L	1.12(0.91–1.34)	1.15(0.91–1.36)	1.09(0.91–1.31)	0.290
LDL, mmol/L	2.51(1.94–3.19)	2.69(2.06–3.40)	2.28(1.82–2.87)	**2.00E**−**04**

^a^Significant *p*-values are in bold. *CHD* coronary heart disease, *HDL* High density lipoprotein, *IQR* interquartile range, *LDL* Low density lipoprotein, *TC* total cholesterol, *TG* triglyceride

### Sample collection and processing

The peripheral whole blood from CHD cases and healthy controls were collected by ethylene diamine tetraacetic acid (EDTA) tubes, and stored at − 80 °C till DNA isolation. Genomic DNA was extracted from peripheral whole blood by the Genomic DNA Extraction Kit (Zymo Research, Orange County, United States). Subsequently, DNA was bisulfite converted with more than 99% efficiency (Additional file 1: Supplementary Fig. 3) by the EZ-96 DNA Methylation Gold Kit according to the standard protocol (Zymo Research, Orange County, United States).

### Matrix-assisted laser desorption/ionization-time-of-flight mass spectrometry

Agena matrix-assisted laser desorption/ionization time-of-flight (MALDI-TOF) mass spectrometry described by Yang et al. [38, 39] was used to quantify DNA methylation within *6p21.33* and *AHRR*. In brief, the bisulfite-converted DNA was amplified by bisulfite-specific primers (no SNPs in the primers), and two PCR amplicons (*6p21.33* amplicon covering 6p21.33_CpG_4/cg06126421 and 4 adjacent measurable CpG sites; *AHRR* amplicon covering AHRR_CpG_3/cg05575921 and 13 adjacent measurable CpG sites) were analyzed. These were obtained with the use of the primers 5’-aggaagagagGGTTGTTGAAAAGGTTAGAAATATAGG-3’ (sense) and 5’-cagtaatacgactcactatagggagaaggctACTATCCCTCCCAACCTTAAAAAA-3’ (antisense) for the *6p21.33* amplicon and primers 5’-aggaagagagGAGGGGTTTTGTTAGGATTATTTTT-3’ (sense) and 5’-cagtaatacgactcactatagggagaaggctAAACCACTCTACTCCAACCCTTACT-3’ (antisense) for the *AHRR* amplicon. Upper case letters present the sequence-specific primer regions, and non-specific tags are shown in lower case letters. The Sequence of the amplicon was presented in Additional file 1: Supplementary Fig. 1. PCR products were treated according to the standard protocol of Agena EpiTyper Assay, and further cleaned by resin, and then dispensed to a 384 SpectroCHIP by a Nanodispenser. The chips were read by a MassARRAY system. Data were collected by EpiTYPER v1.2 software. For each batch of MassARRAY analysis, the same number of CHD cases and controls were treated and analyzed in parallel in all the processes.

### Statistical analyses

The data were analyzed by IBM SPSS Statistics Version 25.0. The measurement data, such as the levels of total cholesterol, total triglyceride, high-density lipoprotein, lowdensity lipoprotein, and the methylation levels of *6p21.33* and *AHRR* are shown as the median (interquartile range (IQR)). The differences between CHD and control subjects were assessed using non-parametric tests (Mann–Whitney U test and Kruskal–Wallis test). Differences in the enumeration data, such as the frequencies of gender, smoking, drinking, hypertension, and diabetes between CHD and control subjects were analyzed using the chi-square (χ^2^) test. Bivariate correlations between variables were examined by Spearman's rank correlation coefficients. Additionally, the logistic regression results with effect ratios (odds ratio (OR) and 95% confidence intervals (CIs)) were adjusted for possible and available confounding effects. Common cardiovascular-related factors, such as TC (< 5.0 mmol/L vs. ≥ 5.0 mmol/L), TG (< 1.7 mmol/L vs. ≥ 1.7 mmol/L), HDL (< 1.0 mmol/L vs. ≥ 1.0 mmol/L) and LDL (< 3.0 mmol/L vs. ≥ 3.0 mmol/L), were divided by general used criteria [40]. Receiver operating characteristic (ROC) curve analysis was applied to assess the discriminatory power of methylation levels. Bonferroni correction was used for the multiple comparisons. The Bonferroni correction was performed by the number of CPG sites in each gene separately. When the corrected *p* value was ≥ 1, it is represented by 1. All the statistical tests were two-sided with *p* values of < 0.05.

## Results

### Association between blood-based *6p21.33* and *AHRR* methylation and CHD

DNA methylation levels at 5 CpG loci (covering cg06126421) of *6p21.33* and 14 CPG sites (covering cg05575921) of *AHRR* were quantitatively determined by mass spectrometry in the blood from 180 CHD patients and 184 controls. The CHD patients have a median age of 66 years old (IQR: 58–73, range from 39 to 87 years old) with 109 males (60.6%) and 71 females (39.4%) (Table 1). Since the controls were recruited from the health examination center where most participants were under 70 years old, our control group was a bit younger than the CHD cases (median of age: 63, IQR: 57–68, range from 41 to 88 years old) with 114 males (62.0%) and 70 females (38.0%) (Table 1). Compared with controls, CHD patients had higher prevalence of hypertension (72.2% vs. 45.7%, *p* = 2 × 10^−6^) and more smokers (40.6% vs. 28.8%, *p* = 0.027). CHD patients had lower TC level (3.81 vs. 4.26 mmol/L, *p* = 0.001) and lower LDL level (2.28 vs. 2.69 mmol/L, *p* = 2 × 10^−4^) than controls (Table 1). The associations between *6p21.33* and *AHRR* methylation and the status of CHD were investigated by two logistic regression models adjusted for different covariants (Table 2). Of which, age, gender, and batch effect were adjusted in model 1, and all the baseline characteristics that had significant differences between the CHD cases and the controls (as shown in Table 1) were adjusted in the logistic regression model 2. With multiple testing corrections, 6p21.33_CpG_4.5/cg06126421 methylation was significantly associated with CHD in model 1 (*p* = 0.032 after Bonferroni correction) but not anymore in the more stringent model 2 (Table 2). None of the 14 measurable CpG loci in *AHRR* displayed any association with CHD in both model 1 and model 2 (Table 2). We also noticed that the methylation correlates better among close than among more distant CpGs. More specific, the methylation correlates better among CpGs in the same amplicon than CpGs in different amplicons which have larger distance. In addition, all CpG sites in the *AHRR* amplicon are positively correlated with each other, while partial CpG sites in the *6p21.33* amplicon are positively, or negatively correlated with each other (Additional file 1: Supplementary Fig. 2).

**Table 2:** Methylation difference of *6p21.33* and *AHRR* comparing CHD cases and controls

CpG sites	Controls (N = 184)	CHD cases (N = 180)	OR (95%CI) per-10%methylation	*p-*value^a^	*p-*value*	OR (95%CI) per-10%methylation	*p-*value^b^	*p-*value#
	Median (IQR)	Median (IQR)						
6p21.33_CpG_1	1.00(0.99–1.00)	1.00(0.98–1.00)	0.96(0.59–1.54)	0.852	1.000	1.01(0.60–1.68)	0.976	1.000
6p21.33_CpG_2	0.93(0.90–0.95)	0.94(0.91–0.97)	0.59(0.36–0.94)	**0.027**	0.108	0.64(0.39–1.06)	0.083	0.332
6p21.33_CpG_3	0.63(0.57–0.70)	0.62(0.54–0.70)	1.11(0.94–1.31)	0.235	1.000	1.14(0.95–1.37)	0.152	0.608
6p21.33_CpG_4.5/cg06126421	0.50(0.45–0.55)	0.48(0.42–0.52)	1.42(1.10–1.83)	**0.008**	**0.032**	1.40(1.07–1.83)	**0.016**	0.064
AHRR_CpG_1	0.73(0.63–0.80)	0.74(0.58–0.83)	1.01(0.90–1.14)	0.846	1.000	1.00(0.87–1.14)	0.959	1.000
AHRR_CpG_2	0.87(0.74–0.96)	0.89(0.72–1.00)	1.02(0.90–1.16)	0.720	1.000	1.15(0.82–1.60)	0.419	1.000
AHRR_CpG_3/cg05575921	0.77(0.64–0.83)	0.75(0.59–0.85)	1.08(0.96–1.22)	0.201	1.000	1.02(0.86–1.20)	0.866	1.000
AHRR_CpG_4.5	0.77(0.68–0.84)	0.75(0.60–0.84)	1.12(0.99–1.27)	0.075	0.750	1.07(0.77–1.49)	0.696	1.000
AHRR_CpG_6	0.84(0.74–0.90)	0.82(0.65–0.91)	1.10(0.96–1.26)	0.177	1.000	1.03(0.89–1.18)	0.691	1.000
AHRR_CpG_7	0.66(0.53–0.76)	0.66(0.53–0.79)	1.01(0.89–1.16)	0.836	1.000	1.07(0.94–1.22)	0.341	1.000
AHRR_CpG_8.9	0.85(0.73–0.94)	0.87(0.76–0.94)	0.89(0.73–1.09)	0.270	1.000	1.15(1.00–1.32)	0.052	0.520
AHRR_CpG_10.11	0.92(0.89–0.95)	0.92(0.87–0.96)	1.25(0.91–1.71)	0.170	1.000	1.08(0.93–1.26)	0.315	1.000
AHRR_CpG_12	0.88(0.81–0.95)	0.88(0.78–0.98)	1.07(0.91–1.25)	0.420	1.000	0.99(0.85–1.14)	0.846	1.000
AHRR_CpG_14.15	0.94(0.91–0.95)	0.94(0.90–0.97)	1.08(0.80–1.47)	0.618	1.000	0.89(0.72–1.09)	0.260	1.000

^a^Model 1: Logistic regression adjusted for age, gender, and batch effect

^b^Model 2: Logistic regression adjusted for age, gender, smoking, hypertension, TC, LDL, and batch effect. Significant *p*-values are in bold

*AHRR* aryl-hydrocarbon receptor repressor, *CI* confidence interval, *CpG* cytidine-phosphate-guanosine, *OR* odds ratio. *^, #^Bonferroni-corrected *p* values. Bold values indicated *p* < 0.05

### Methylation difference of *6p21.33* and *AHRR* between patients with heart failure, MI, non-MI CHD, and controls

Of the 180 CHD patients, 145 suffered from heart failure, 78 had experienced MI, and 102 were non-MI CHD cases. We further investigated the association between these CHD subtypes and the blood-based methylation of *6p21.33* and *AHRR* also by the two logistic regression models adjusted for different covariants (Table 3). Compared with the healthy controls, heart failure CHD patients have significantly decreased methylation at 6p21.33_CpG_4.5/cg06126421 by both logistic regression model 1 and model 2 (model 1: OR per − 10% methylation (95% CI) = 1.62 (1.21–2.17), *p* = 0.004 after Bonferroni correction; model 2: OR per − 10% methylation (95% CI) = 1.59 (1.17–2.16), *p* = 0.012 after Bonferroni correction, Panel A of Table 3). Since there are only 35 CHD patients without heart failure, the Mann–Whitney U test was applied to assess the *6p21.33* and *AHRR* methylation difference between the non-heart failure CHD cases and controls and found no significant differences (Additional file 2: Table S1).

**Table 3:** Methylation difference of *6p21.33* and *AHRR* comparing heart failure cases, MI cases, non-MI CHD cases, and controls

CpG sites	Controls (N = 184)	Heart failure cases (N = 145)	OR (95%CI) per-10%methylation	*p-*value^a^	*p-*value*	OR (95%CI) per-10%methylation	*p-*value^b^	*p-*value#
	Median (IQR)	Median (IQR)						
*Panel (A). Heart failure cases vs. Controls*								
6p21.33_CpG_1	1.00(0.99–1.00)	1.00(0.99–1.00)	0.75(0.44–1.30)	0.308	1.000	0.78(0.44–1.38)	0.398	1.000
6p21.33_CpG_2	0.93(0.90–0.95)	0.94(0.91–0.96)	0.52(0.30–0.89)	**0.017**	0.068	0.56(0.31–0.98)	**0.044**	0.176
6p21.33_CpG_3	0.63(0.57–0.70)	0.61(0.53–0.69)	1.16(0.97–1.40)	0.113	0.452	1.19(0.98–1.45)	0.079	0.316
6p21.33_CpG_4.5/cg06126421	0.50(0.45–0.55)	0.48(0.42–0.52)	1.62(1.21–2.17)	**0.001**	**0.004**	1.59(1.17–2.16)	**0.003**	**0.012**
AHRR_CpG_1	0.73(0.63–0.80)	0.74(0.56–0.82)	1.04(0.91–1.18)	0.607	1.000	1.02(0.89–1.18)	0.775	1.000
AHRR_CpG_2	0.87(0.74–0.96)	0.88(0.70–1.00)	1.08(0.94–1.24)	0.267	1.000	1.10(0.94–1.28)	0.246	1.000
AHRR_CpG_3/cg05575921	0.77(0.64–0.83)	0.75(0.59–0.84)	1.09(0.96–1.24)	0.200	1.000	1.08(0.93–1.24)	0.319	1.000
AHRR_CpG_4.5	0.77(0.68–0.84)	0.74(0.59–0.84)	1.16(1.01–1.34)	**0.031**	0.310	1.19(1.02–1.38)	**0.024**	0.240
AHRR_CpG_6	0.84(0.74–0.90)	0.82(0.65–0.91)	1.12(0.96–1.30)	0.140	1.000	1.11(0.94–1.32)	0.217	1.000
AHRR_CpG_7	0.66(0.53–0.76)	0.67(0.54–0.82)	1.05(0.91–1.21)	0.529	1.000	1.02(0.87–1.18)	0.850	1.000
AHRR_CpG_8.9	0.85(0.73–0.94)	0.89(0.77–0.95)	0.95(0.77–1.18)	0.664	1.000	0.93(0.74–1.17)	0.542	1.000
AHRR_CpG_10.11	0.92(0.89–0.95)	0.92(0.88–0.95)	1.07(0.70–1.63)	0.762	1.000	0.96(0.61–1.51)	0.855	1.000
AHRR_CpG_12	0.88(0.81–0.95)	0.88(0.79–0.97)	1.03(0.85–1.24)	0.793	1.000	0.97(0.80–1.19)	0.798	1.000
AHRR_CpG_14.15	0.94(0.91–0.95)	0.94(0.90–0.97)	1.03(0.75–1.43)	0.841	1.000	1.04(0.74–1.45)	0.828	1.000

CpG sites	Controls (N = 184)	MI cases (N = 78)	OR (95%CI) per-10%methylation	*p-*value^a^	*p-*value*	OR (95%CI) per-10%methylation	*p-*value^b^	*p-*value#
	Median (IQR)	Median (IQR)						
*Panel (B). MI cases vs. Controls*								
6p21.33_CpG_1	1.00(0.99–1.00)	1.00(0.97–1.00)	1.60(0.87–2.92)	0.128	0.512	1.54(0.83–2.87)	0.173	0.692
6p21.33_CpG_2	0.93(0.90–0.95)	0.94(0.89–0.97)	0.64(0.35–1.18)	0.156	0.624	0.64(0.34–1.21)	0.170	0.680
6p21.33_CpG_3	0.63(0.57–0.70)	0.62(0.53–0.70)	1.17(0.95–1.44)	0.150	0.600	1.17(0.94–1.45)	0.161	0.644
6p21.33_CpG_4.5/cg06126421	0.50(0.45–0.55)	0.48(0.40–0.51)	1.45(1.04–2.02)	**0.030**	0.120	1.42(1.01–1.99)	**0.042**	0.168
AHRR_CpG_1	0.73(0.63–0.80)	0.68(0.53–0.82)	1.07(0.92–1.24)	0.411	1.000	1.05(0.89–1.23)	0.584	1.000
AHRR_CpG_2	0.87(0.74–0.96)	0.84(0.62–0.98)	1.07(0.92–1.25)	0.394	1.000	1.06(0.90–1.25)	0.505	1.000
AHRR_CpG_3/cg05575921	0.77(0.64–0.83)	0.72(0.50–0.84)	1.10(0.96–1.27)	0.179	1.000	1.08(0.92–1.25)	0.344	1.000
AHRR_CpG_4.5	0.77(0.68–0.84)	0.74(0.54–0.83)	1.15(0.99–1.35)	0.073	0.730	1.15(0.98–1.36)	0.093	0.930
AHRR_CpG_6	0.84(0.74–0.90)	0.79(0.61–0.91)	1.15(0.97–1.35)	0.100	1.000	1.12(0.94–1.33)	0.207	1.000
AHRR_CpG_7	0.66(0.53–0.76)	0.61(0.49–0.73)	1.06(0.89–1.26)	0.526	1.000	1.03(0.86–1.23)	0.778	1.000
AHRR_CpG_8.9	0.85(0.73–0.94)	0.82(0.69–0.92)	0.99(0.77–1.29)	0.966	1.000	0.97(0.75–1.27)	0.838	1.000
AHRR_CpG_10.11	0.92(0.89–0.95)	0.92(0.85–0.96)	1.47(1.01–2.14)	**0.042**	**0.420**	1.39(0.95–2.04)	0.091	0.910
AHRR_CpG_12	0.88(0.81–0.95)	0.87(0.75–1.00)	1.12(0.92–1.37)	0.245	1.000	1.08(0.88–1.32)	0.450	1.000
AHRR_CpG_14.15	0.94(0.91–0.95)	0.94(0.87–0.97)	1.38(0.84–2.27)	0.210	1.000	1.41(0.83–2.38)	0.203	1.000

CpG sites	Controls (N = 184)	Non-MI CHD cases (N = 102)	OR (95%CI) per-10%methylation	*p-*value^a^	*p-*value*	OR (95%CI) per-10%methylation	*p-*value^b^	*p-*value#
	Median (IQR)	Median (IQR)						
*Panel (C). Non-MI CHD cases vs. Controls*								
6p21.33_CpG_1	1.00(0.99–1.00)	1.00(0.99–1.00)	0.54(0.28–1.04)	0.064	0.256	0.56(0.29–1.11)	0.095	0.380
6p21.33_CpG_2	0.93(0.90–0.95)	0.94(0.92–0.97)	0.55(0.29–1.03)	0.061	0.244	0.70(0.35–1.39)	0.302	1.000
6p21.33_CpG_3	0.63(0.57–0.70)	0.62(0.55–0.70)	1.09(0.87–1.37)	0.448	1.000	1.16(0.91–1.48)	0.220	0.880
6p21.33_CpG_4.5/cg06126421	0.50(0.45–0.55)	0.48(0.44–0.52)	1.41(1.01–1.98)	**0.047**	0.188	1.39(0.97–1.99)	0.077	0.308
AHRR_CpG_1	0.73(0.63–0.80)	0.77(0.60–0.83)	0.96(0.81–1.12)	0.581	1.000	0.92(0.77–1.11)	0.400	1.000
AHRR_CpG_2	0.87(0.74–0.96)	0.91(0.76–1.00)	0.99(0.82–1.18)	0.863	1.000	0.99(0.81–1.22)	0.948	1.000
AHRR_CpG_3/cg05575921	0.77(0.64–0.83)	0.77(0.62–0.85)	1.06(0.90–1.25)	0.456	1.000	1.09(0.90–1.31)	0.373	1.000
AHRR_CpG_4.5	0.77(0.68–0.84)	0.76(0.62–0.85)	1.14(0.96–1.34)	0.128	1.000	1.20(0.99–1.45)	0.059	0.590
AHRR_CpG_6	0.84(0.74–0.90)	0.84(0.71–0.91)	1.05(0.87–1.26)	0.641	1.000	1.03(0.83–1.29)	0.774	1.000
AHRR_CpG_7	0.66(0.53–0.76)	0.70(0.57–0.83)	0.97(0.82–1.15)	0.753	1.000	0.95(0.78–1.14)	0.567	1.000
AHRR_CpG_8.9	0.85(0.73–0.94)	0.89(0.79–0.96)	0.81(0.62–1.05)	0.115	1.000	0.80(0.60–1.06)	0.112	1.000
AHRR_CpG_10.11	0.92(0.89–0.95)	0.92(0.88–0.96)	1.10(0.69–1.76)	0.680	1.000	1.00(0.61–1.65)	0.988	1.000
AHRR_CpG_12	0.88(0.81–0.95)	0.89(0.80–0.96)	1.07(0.86–1.34)	0.550	1.000	1.04(0.81–1.33)	0.770	1.000
AHRR_CpG_14.15	0.94(0.91–0.95)	0.94(0.92–0.97)	1.01(0.70–1.44)	0.968	1.000	1.00(0.69–1.43)	0.984	1.000

^a^Model 1: Logistic regression adjusted for age, gender, and batch effect

^b^Model 2: Logistic regression adjusted for age, gender, smoking, hypertension, TC, LDL, and batch effect. Significant *p*-values are in bold. *MI* myocardial infarction. *^,#^Bonferroni-corrected *p* values. Bold values indicated *p* < 0.05

Next, we assessed the association between the methylation of *6p21.33* and *AHRR* and the status of MI. The decreased methylation for the MI cases compared to the controls was also detected in the 6p21.33_CpG_4.5/cg06126421 by both model 1 and model 2, but not significant after Bonferroni correction (Panel B of Table 3). All the 14 *AHRR* CpG sites showed no association with MI by both models (Panel B of Table 3). In addition, none of the 19 measurable CpG sites in *6p21.33* and *AHRR* showed any association with the non-MI CHD cases by the two logistic regression models (Panel C of Table 3).

### Association between blood-based *6p21.33* and *AHRR* methylation and early CHD cases

In our study, the cardiac function of 124 CHD cases was classified as NYHA I and NYHA II (NYHA I CHD cases = 46, NYHA II CHD cases = 78). Compared to the healthy controls, the methylation intensity of *6p21.33* at the 6p21.33_CpG_4.5/cg06126421 locus was also significantly decreased in NYHA I&II CHD cases by the two logistic regression models (model 1: OR per − 10% methylation (95% CI) = 1.69 (1.22–2.34), *p* = 0.008 after Bonferroni correction; model 2: OR per − 10% methylation (95% CI) = 1.65 (1.17–2.34), *p* = 0.020 after Bonferroni correction, Table 4). No significant association between the early-stage cardiovascular dysfunction cases and the methylation changes was observed for all the 14 CpG sites of *AHRR* (Table 4). All the five CpG sites in *6p21.33* and half of the CpG sites in *AHRR* had lower methylation levels in the 37 NYHA III&IV CHD cases than that in the 124 NYHA I&II CHD cases but without significance probably due to the limited sample size (Additional file 1: Table 2). Nevertheless, these observations indicated that the aberrant blood-based DNA methylation might be enhanced along with the progress of cardiac dysfunction.

**Table 4:** Methylation difference of *6p21.33* and *AHRR* comparing NYHA I&II CHD cases and controls

CpG sites	Controls (N = 184)	NYHA I&II CHD cases (N = 124)	OR (95%CI) per-10%methylation	*p-*value^a^	*p-*value*	OR (95%CI) per-10%methylation	*p-*value^b^	*p-*value#
	Median (IQR)	Median (IQR)						
6p21.33_CpG_1	1.00(0.99–1.00)	1.00(1.00–1.00)	0.79(0.49–1.27)	0.328	1.000	0.78(0.48–1.27)	0.315	1.000
6p21.33_CpG_2	0.93(0.90–0.95)	0.94(0.91–0.97)	0.49(0.28–0.85)	**0.011**	**0.044**	0.54(0.30–0.97)	**0.038**	0.152
6p21.33_CpG_3	0.63(0.57–0.70)	0.62(0.53–0.69)	1.15(0.94–1.41)	0.164	0.656	1.21(0.98–1.50)	0.079	0.316
6p21.33_CpG_4.5/cg06126421	0.50(0.45–0.55)	0.49(0.44–0.52)	1.69(1.22–2.34)	**0.002**	**0.008**	1.65(1.17–2.34)	**0.005**	**0.020**
AHRR_CpG_1	0.73(0.63–0.80)	0.74(0.59–0.82)	1.04(0.90–1.20)	0.592	1.000	1.03(0.88–1.21)	0.736	1.000
AHRR_CpG_2	0.87(0.74–0.96)	0.89(0.72–1.00)	1.04(0.89–1.21)	0.618	1.000	1.06(0.89–1.26)	0.508	1.000
AHRR_CpG_3/cg05575921	0.77(0.64–0.83)	0.75(0.60–0.85)	1.10(0.95–1.26)	0.201	1.000	1.11(0.94–1.30)	0.211	1.000
AHRR_CpG_4.5	0.77(0.68–0.84)	0.76(0.61–0.84)	1.13(0.97–1.31)	0.115	1.000	1.16(0.98–1.37)	0.081	0.810
AHRR_CpG_6	0.84(0.74–0.90)	0.83(0.68–0.90)	1.11(0.94–1.31)	0.239	1.000	1.10(0.91–1.33)	0.316	1.000
AHRR_CpG_7	0.66(0.53–0.76)	0.69(0.55–0.82)	1.02(0.88–1.19)	0.772	1.000	1.01(0.85–1.19)	0.930	1.000
AHRR_CpG_8.9	0.85(0.73–0.94)	0.89(0.78–0.94)	0.92(0.74–1.16)	0.488	1.000	0.91(0.72–1.15)	0.436	1.000
AHRR_CpG_10.11	0.92(0.89–0.95)	0.92(0.88–0.95)	1.02(0.65–1.60)	0.929	1.000	0.93(0.57–1.51)	0.756	1.000
AHRR_CpG_12	0.88(0.81–0.95)	0.89(0.80–0.95)	1.05(0.86–1.29)	0.620	1.000	1.03(0.83–1.28)	0.792	1.000
AHRR_CpG_14.15	0.94(0.91–0.95)	0.94(0.91–0.96)	1.08(0.77–1.50)	0.670	1.000	1.09(0.76–1.57)	0.649	1.000

^a^Model 1: Logistic regression adjusted for age, gender, and batch effect\

^b^Model 2: Logistic regression adjusted for age, gender, smoking, hypertension, TC, LDL, and batch effect. Significant *p* values are in bold. *NYHA* New York Heart Association. *^, #^Bonferroni-corrected *p* values. Bold values indicated *p* < 0.05

### The correlation between blood-based *6p21.33* and *AHRR* methylation and CHD-related characteristics

Methylation intensities across various strata of the CHD cases and controls respectively were shown in Table 5 for *6p21.33* cg06126421, *AHRR* cg05575921, and their adjacent measurable CpG sites. In agreement with previous reports [36, 41–43], smokers had significantly lower *AHRR* methylation than the non-smokers in both controls and CHD cases (Table 5). In opposite, the smoking-related lower *6p21.33* methylation was significant only in CHD cases but not in the controls (Table 5). Our results also suggested drinking as a causative factor for the hypomethylation of *AHRR* mostly in controls*,* but such association is much weaker than smoking (Table 5). Drinkers showed no significant *6p21.33* methylation changes compared with non-drinkers in both controls and CHD cases. Compared to the women, men had lower methylation in *6p21.33* and *AHRR* in both controls and CHD cases (Table 5). Our results showed no or very weak *6p21.33* and *AHRR* methylation difference among people with the variant status of hypertension and diabetes, and people with different levels of TC, TG, HDL, and LDL (Table 5).

**Table 5:** The association between *6p21.33* and *AHRR* methylation and CHD-related characteristics in CHD patients and Controls

Characteristics	Group (N)	Median of methylation intensity													
		6p21.33_CpG_1	6p21.33_CpG_2	6p21.33_CpG_3	6p21.33_CpG_4.5/cg06126421	AHRR_CpG_1	AHRR_CpG_2	AHRR_CpG_3/cg05575921	AHRR_CpG_4.5	AHRR_CpG_6	AHRR_CpG_7	AHRR_CpG_8.9	AHRR_CpG_10.11	AHRR_CpG_12	AHRR_CpG_14.15
*CHD cases*															
Age (180)	< 65 (83)	1.00	0.94	0.64	0.48	0.77	0.90	0.75	0.74	0.82	0.66	0.87	0.92	0.88	0.94
	≥ 65 (97)	1.00	0.94	0.61	0.48	0.69	0.89	0.76	0.76	0.82	0.66	0.87	0.92	0.89	0.94
	*p*-value^a^	0.894	0.998	0.143	1.000	0.050	0.617	0.875	0.812	0.661	0.583	0.624	0.166	0.884	0.870
Gender (180)	Female (71)	1.00	0.94	0.65	0.49	0.78	0.97	0.81	0.79	0.88	0.71	0.88	0.93	0.91	0.94
	Male (109)	1.00	0.93	0.60	0.47	0.66	0.83	0.70	0.73	0.76	0.63	0.86	0.91	0.86	0.94
	*p*-value^a^	0.646	0.136	**0.009**	0.050	**0.003**	**1.00E**−**06**	**1.00E**−**05**	**0.003**	**7.60E**−**05**	**0.039**	0.206	0.148	**0.039**	0.144
Smoking (180)	No (107)	1.00	0.94	0.63	0.49	0.78	0.94	0.80	0.78	0.86	0.70	0.86	0.93	0.90	0.94
	Yes (73)	1.00	0.94	0.59	0.45	0.63	0.76	0.62	0.65	0.71	0.61	0.87	0.90	0.84	0.94
	*p*-value^a^	0.372	0.615	**0.017**	**0.005**	**0.001**	**2.00E**−**06**	**2.15E**−**07**	**0.001**	**3.20E**−**05**	**0.011**	0.381	**0.001**	**0.028**	0.483
Drinking (180)	No (128)	1.00	0.93	0.62	0.49	0.75	0.91	0.77	0.76	0.83	0.66	0.86	0.92	0.89	0.94
	Yes (52)	1.00	0.95	0.62	0.48	0.64	0.84	0.64	0.73	0.76	0.66	0.89	0.91	0.86	0.94
	*p*-value^a^	0.075	0.189	0.560	0.332	0.127	0.063	**0.011**	0.321	0.101	0.706	0.580	0.227	0.465	0.575
Hypertension (180)	Not prevalent (50)	1.00	0.93	0.62	0.47	0.70	0.84	0.73	0.73	0.78	0.61	0.85	0.92	0.91	0.95
	Prevalent (130)	1.00	0.94	0.62	0.49	0.74	0.89	0.76	0.76	0.83	0.66	0.87	0.92	0.88	0.94
	*p*-value^a^	0.345	0.117	0.166	0.318	0.375	0.170	0.592	0.102	0.154	0.332	0.280	0.742	0.428	0.459
Diabetes (180)	Not prevalent (118)	1.00	0.93	0.61	0.49	0.74	0.88	0.74	0.74	0.80	0.64	0.85	0.92	0.86	0.94
	Prevalent (62)	1.00	0.95	0.63	0.47	0.73	0.92	0.77	0.76	0.84	0.72	0.89	0.92	0.89	0.95
	*p*-value^a^	0.589	0.064	0.300	0.699	0.908	0.119	0.191	0.343	0.341	**0.021**	0.095	0.392	0.376	0.422
TC (180)	< 5.0 mmol/L (153)	1.00	0.94	0.62	0.48	0.72	0.89	0.74	0.74	0.81	0.64	0.87	0.92	0.88	0.94
	≥ 5.0 mmol/L (27)	1.00	0.93	0.64	0.48	0.77	0.93	0.77	0.76	0.89	0.72	0.86	0.92	0.90	0.94
	*p*-value^a^	0.998	0.748	0.732	0.905	0.286	0.355	0.198	0.739	0.085	0.283	0.823	0.542	0.166	0.594
TG (180)	< 1.70 mmol/L (123)	1.00	0.94	0.62	0.48	0.74	0.89	0.77	0.74	0.82	0.66	0.87	0.92	0.88	0.94
	≥ 1.70 mmol/L (57)	1.00	0.93	0.62	0.48	0.72	0.89	0.73	0.76	0.83	0.66	0.86	0.92	0.89	0.95
	*p*-value^a^	0.558	0.999	0.843	0.775	0.841	0.904	0.356	0.304	0.463	0.763	0.255	0.762	0.900	0.268
HDL (180)	< 1.0 mmol/L (67)	1.00	0.94	0.60	0.46	0.72	0.87	0.73	0.73	0.77	0.64	0.86	0.92	0.87	0.94
	≥ 1.0 mmol/L (113)	1.00	0.94	0.63	0.49	0.74	0.90	0.76	0.76	0.84	0.67	0.87	0.92	0.89	0.94
	*p*-value^a^	0.466	0.691	0.124	0.236	0.951	0.194	0.347	0.099	**0.034**	0.522	0.523	0.295	0.086	0.686
LDL (180)	< 3.0 mmol/L (143)	1.00	0.94	0.61	0.48	0.73	0.89	0.75	0.74	0.81	0.64	0.87	0.92	0.88	0.94
	≥ 3.0 mmol/L (37)	1.00	0.93	0.65	0.49	0.76	0.89	0.77	0.76	0.89	0.68	0.86	0.92	0.89	0.94
	*p*-value^a^	0.417	0.924	0.145	0.296	0.366	0.336	0.991	0.208	0.083	0.383	0.935	0.745	0.098	0.456
*Controls*															
Age (184)	< 65 (119)	1.00	0.93	0.64	0.50	0.73	0.87	0.76	0.77	0.82	0.69	0.87	0.92	0.88	0.93
	≥ 65 (65)	1.00	0.93	0.62	0.50	0.74	0.86	0.77	0.76	0.84	0.61	0.79	0.93	0.89	0.94
	*p*-value^a^	0.326	0.931	0.297	0.572	0.954	0.546	0.964	0.598	0.978	**0.027**	**0.016**	0.973	0.916	0.105
Gender (184)	Female (70)	1.00	0.93	0.67	0.53	0.77	0.92	0.79	0.80	0.85	0.66	0.80	0.93	0.92	0.94
	Male (114)	1.00	0.92	0.62	0.49	0.70	0.83	0.74	0.74	0.81	0.66	0.87	0.92	0.87	0.93
	*p*-value^a^	0.052	0.130	**0.004**	**4.17E**−**04**	**0.001**	**2.00E**−**06**	**0.008**	**0.002**	**0.048**	0.815	0.128	0.284	**0.004**	0.672
Smoking (180)	No (127)	1.00	0.93	0.64	0.51	0.76	0.89	0.79	0.78	0.85	0.68	0.85	0.93	0.90	0.94
	Yes (53)	1.00	0.92	0.62	0.48	0.62	0.75	0.67	0.69	0.76	0.59	0.85	0.91	0.85	0.93
	*p*-value^a^	0.526	0.916	0.684	0.065	**2.00E**−**06**	**1.00E-06**	**1.22E-04**	**0.001**	**0.001**	0.063	0.911	**0.019**	**0.010**	0.132
Drinking (180)	No (114)	1.00	0.93	0.63	0.50	0.76	0.88	0.78	0.78	0.84	0.63	0.80	0.92	0.89	0.94
	Yes (66)	1.00	0.93	0.64	0.51	0.69	0.85	0.73	0.75	0.82	0.71	0.90	0.93	0.88	0.94
	*p*-value^a^	0.508	0.982	0.450	0.422	**0.010**	0.084	0.060	0.318	0.308	**0.030**	**0.002**	0.391	0.283	0.687
Hypertension (177)	Not prevalent (93)	1.00	0.92	0.62	0.50	0.75	0.87	0.77	0.76	0.85	0.68	0.86	0.92	0.88	0.93
	Prevalent (84)	1.00	0.93	0.65	0.50	0.72	0.85	0.75	0.77	0.82	0.63	0.82	0.92	0.88	0.94
	*p*-value^a^	0.879	0.343	0.608	0.745	0.819	0.972	0.732	0.776	0.336	0.465	0.810	0.735	0.806	0.336
Diabetes (177)	Not prevalent (132)	1.00	0.92	0.64	0.50	0.74	0.87	0.78	0.77	0.84	0.65	0.82	0.92	0.89	0.93
	Prevalent (45)	1.00	0.93	0.62	0.51	0.72	0.85	0.73	0.75	0.82	0.69	0.88	0.92	0.87	0.94
	*p*-value^a^	0.796	**0.043**	0.719	0.769	0.802	0.356	0.217	0.616	0.514	0.721	0.402	0.653	0.434	0.528
TC (180)	< 5.0 mmol/L (131)	1.00	0.93	0.64	0.50	0.74	0.86	0.76	0.76	0.83	0.65	0.84	0.93	0.89	0.94
	≥ 5.0 mmol/L (49)	1.00	0.93	0.62	0.51	0.70	0.87	0.75	0.78	0.84	0.69	0.88	0.92	0.87	0.92
	*p*-value^a^	0.992	0.464	0.959	0.506	0.698	0.516	0.524	0.559	0.930	0.798	0.079	0.586	0.107	**0.001**
TG (178)	< 1.70 mmol/L (114)	1.00	0.93	0.62	0.50	0.75	0.87	0.78	0.78	0.84	0.65	0.83	0.93	0.90	0.94
	≥ 1.70 mmol/L (64)	1.00	0.93	0.65	0.50	0.70	0.85	0.73	0.75	0.83	0.70	0.88	0.92	0.87	0.93
	*p*-value^a^	**0.018**	0.755	0.891	0.759	0.482	0.434	**0.037**	0.128	0.647	0.174	0.137	0.781	0.157	0.332
HDL (180)	< 1.0 mmol/L (63)	1.00	0.93	0.62	0.50	0.70	0.85	0.73	0.75	0.81	0.69	0.87	0.92	0.87	0.94
	≥ 1.0 mmol/L (117)	1.00	0.93	0.64	0.51	0.75	0.88	0.77	0.78	0.85	0.65	0.85	0.93	0.90	0.93
	*p*-value^a^	0.440	0.735	0.260	0.375	0.429	0.170	0.120	0.135	0.185	0.420	0.279	0.866	0.334	0.112
LDL (180)	< 3.0 mmol/L (108)	1.00	0.93	0.64	0.51	0.73	0.85	0.75	0.76	0.84	0.65	0.85	0.93	0.88	0.94
	≥ 3.0 mmol/L (72)	1.00	0.93	0.62	0.50	0.75	0.87	0.79	0.78	0.82	0.67	0.87	0.92	0.89	0.93
	*p*-value^a^	0.822	0.720	0.651	0.572	0.655	0.345	0.659	0.626	0.731	0.966	0.231	0.635	0.957	**0.021**

^a^The *p*-values were calculated by the Mann–Whitney test, and significant *p* values are in bold

Most CHD patients have a history of medication. It seems that the intake of aspirin could significantly reverse the demethylation of *6p21.33* in the blood of CHD cases, but had no influence on the *AHRR* methylation (Table 6). The intake of digoxin was weakly correlated with the hypomethylation of two *AHRR* CpG sites (Table 6). The other 10 common cardiovascular drugs showed no obvious influence on the methylation intensities of *6p21.33* and *AHRR* in the blood of CHD patients (Table 6).

**Table 6:** The methylation of *6p21.33* and *AHRR* in CHD patients with variant medical treatments

Medicine	Group (N)	Median of methylation intensity													
		6p21.33_CpG_1	6p21.33_CpG_2	6p21.33_CpG_3	6p21.33_CpG_4.5/cg06126421	AHRR_CpG_1	AHRR_CpG_2	AHRR_CpG_3/cg05575921	AHRR_CpG_4.5	AHRR_CpG_6	AHRR_CpG_7	AHRR_CpG_8.9	AHRR_CpG_10.11	AHRR_CpG_12	AHRR_CpG_14.15
ACEI	No (147)	1.00	0.94	0.62	0.49	0.73	0.90	0.75	0.76	0.83	0.67	0.87	0.92	0.87	0.94
	Yes (33)	1.00	0.95	0.61	0.45	0.78	0.77	0.77	0.66	0.77	0.63	0.84	0.92	0.91	0.94
	*p*-value*	0.334	0.411	0.660	0.134	0.723	**0.035**	0.988	0.092	0.278	0.050	0.343	0.474	0.297	0.543
ARB	No (139)	1.00	0.93	0.62	0.48	0.74	0.88	0.76	0.75	0.82	0.64	0.87	0.92	0.89	0.94
	Yes (41)	1.00	0.95	0.64	0.50	0.71	0.90	0.73	0.75	0.79	0.71	0.86	0.92	0.85	0.93
	*p*-value*	0.417	0.319	0.278	0.111	0.412	0.880	0.349	0.892	0.539	0.154	0.996	0.369	0.171	0.342
CCB	No (126)	1.00	0.94	0.63	0.48	0.74	0.89	0.77	0.75	0.83	0.67	0.87	0.91	0.88	0.94
	Yes (54)	1.00	0.94	0.60	0.49	0.69	0.89	0.74	0.76	0.81	0.64	0.85	0.92	0.88	0.94
	*p*-value*	0.701	0.414	**0.040**	0.812	0.557	0.641	0.106	0.980	0.710	0.816	0.666	0.359	0.844	0.994
β blocker	No (64)	1.00	0.94	0.61	0.48	0.71	0.92	0.77	0.76	0.81	0.67	0.88	0.92	0.88	0.94
	Yes (116)	1.00	0.94	0.63	0.48	0.74	0.88	0.75	0.74	0.82	0.64	0.86	0.92	0.88	0.94
	*p*-value*	0.352	0.328	0.651	0.845	0.492	0.294	0.622	0.684	0.813	0.263	0.612	0.948	0.664	0.271
Spironolactone	No (143)	1.00	0.93	0.62	0.48	0.74	0.89	0.75	0.75	0.82	0.67	0.87	0.92	0.87	0.94
	Yes (37)	1.00	0.94	0.64	0.49	0.75	0.88	0.76	0.74	0.82	0.59	0.84	0.92	0.91	0.96
	*p*-value*	0.262	0.128	0.696	0.775	0.971	0.497	0.355	0.685	0.913	0.225	0.055	0.054	0.069	0.061
Digoxin	No (169)	1.00	0.94	0.62	0.48	0.74	0.89	0.75	0.75	0.82	0.66	0.87	0.92	0.89	0.94
	Yes (11)	1.00	0.91	0.59	0.45	0.52	0.71	0.71	0.60	0.63	0.52	0.80	0.92	0.84	0.91
	*p*-value*	0.894	0.130	0.475	0.307	0.087	**0.044**	0.605	0.093	0.311	**0.045**	0.063	0.565	0.521	0.341
Nitrates	No (84)	1.00	0.93	0.61	0.48	0.69	0.88	0.72	0.74	0.80	0.63	0.87	0.92	0.88	0.94
	Yes (96)	1.00	0.94	0.63	0.49	0.75	0.89	0.76	0.75	0.83	0.69	0.87	0.92	0.88	0.94
	*p*-value*	0.878	0.706	0.295	0.408	0.230	0.272	0.184	0.760	0.458	0.066	0.891	0.730	0.878	0.918
Aspirin	No (42)	1.00	0.93	0.60	0.45	0.69	0.92	0.77	0.74	0.81	0.68	0.89	0.93	0.88	0.96
	Yes (136)	1.00	0.94	0.63	0.49	0.74	0.89	0.75	0.75	0.82	0.66	0.87	0.92	0.88	0.94
	*p*-value*	0.590	0.509	**0.044**	**0.044**	0.666	0.902	0.363	0.706	0.716	0.683	0.599	0.194	0.930	**0.007**
Clopidogrel	No (79)	1.00	0.94	0.61	0.48	0.74	0.90	0.75	0.76	0.79	0.63	0.87	0.92	0.88	0.94
	Yes (101)	1.00	*0.93*	0.63	0.48	0.73	0.88	0.76	0.74	0.84	0.67	0.86	0.92	0.89	0.94
	*p*-value*	0.156	*0.535*	0.100	0.380	0.953	0.871	0.775	0.985	0.256	0.768	0.971	0.999	0.544	0.573
Warfarin	No (175)	1.00	0.94	0.62	0.48	0.74	0.89	0.75	0.75	0.82	0.66	0.87	0.92	0.88	0.94
	Yes (5)	1.00	0.93	0.64	0.47	0.72	0.77	0.66	0.63	0.78	0.54	0.89	0.91	1.00	0.93
	*p*-value*	0.521	0.386	0.895	0.989	0.923	0.646	0.743	0.844	0.297	0.817	0.489	0.505	0.212	0.920
Statin	No (19)	1.00	0.94	0.58	0.45	0.73	0.93	0.84	0.81	0.84	0.64	0.87	0.91	0.93	0.95
	Yes (161)	1.00	0.94	0.62	0.48	0.74	0.88	0.75	0.74	0.81	0.66	0.87	0.92	0.88	0.94
	*p*-value*	0.510	0.716	0.209	0.131	0.750	0.594	0.121	0.228	0.914	0.696	0.874	0.710	0.658	0.325
Antacids	No (119)	1.00	0.94	0.63	0.49	0.74	0.91	0.76	0.76	0.84	0.66	0.87	0.92	0.89	0.94
	Yes (61)	1.00	0.93	0.61	0.47	0.69	0.88	0.73	0.73	0.79	0.64	0.86	0.92	0.85	0.94
	*p*-value*	0.959	0.525	0.624	0.669	0.515	0.333	0.226	0.328	0.354	0.757	0.824	0.364	0.072	0.341

^*^The *p*-values were calculated by the Mann–Whitney test, and significant *p*-values are in bold

## Discussion

Previous studies have demonstrated that smoking could result in the hypomethylation of *AHRR* cg05575921 and *6p21.33* cg06126421 in the peripheral blood [28, 29]. The association between *AHRR* cg05575921 methylation and cardiovascular disease was also well addressed in Caucasians even in prospective studies [20, 33–36]. In this case–control study, we validated the strong association between smoking and the hypomethylation of *AHRR* cg05575921 and *6p21.33* cg06126421 in the Chinese population. More importantly, we have disclosed the significant hypomethylation of *6p21.33* cg06126421 in the blood leukocyte DNA of CHD patients, especially for CHD patients with heart failure. Our observation agreed with the report of Agha et al. [44] that the blood leukocyte DNA methylation could predict the risk of future MI and CHD. This decreased *6p21.33* methylation could be detected in the blood of patients with early cardiac dysfunction (NYHA I&II CHD cases) and might become more aberrant in the patients with advanced cardiac dysfunction (NYHA III&IV CHD cases). Thus, we proposed that the altered *6p21.33* methylation in the blood leukocytes may play a role not only in the occurrence of CHD but also in the progress.

*6p21.33* cg06126421 is located at chr6:30720081 (build 37/hg19). In the 100 kilobases flanking cg06126421 there are seven genes that code for proteins functioned in the protection of cells from Fas- or tumor necrosis factor type alpha-induced apoptosis (*IER3*), the intra-S phase, and G2/M phase cell cycle checkpoints in response to DNA damage (*MDC1*), cell cycle progression (*DHX16*), cytoskeleton regulation and membrane transport (*FLOT1*, *TUBB*, *PPP1R18*, *NRM*). In particular, the expression of *IER3* is involved in immune functions and the physiology of the cardiovascular system in transgenic and knock-out mouse models [45]. *AHRR* methylation has been associated with CHD in Caucasians, but could not be validated in the Chinese population. Other studies have shown that ethnic genetic background or lifestyle could play a role in epigenetic modifications [46–48]. Thus, DNA methylation patterns warrant validation when the different ethnic population is considered.

In our study, we confirmed the association between the behavior of smoking and the hypomethylation of *AHRR* cg05575921, *6p21.33* cg06126421, and their adjacent CpG sites, and supported the relationship between smoking and epigenetic regulation in atherosclerotic disease [30]. However, in lack of the data on the intensity of the smoking history (pack-years), smoking can hardly be fully adjusted by logistic regression in our study. Future studies with larger samples size and more detailed data on smoking history shall provide more robust evidence for the relationship among smoking, *6p21.33* methylation, and CHD. Our results also showed that the behavior of drinking was associated with the methylation intensity of *AHRR*, but not with the *6p21.33* methylation. This observation was consistent with the reported alcohol-related DNA methylation signatures in specific genes [49]. The incidence of CHD in all age groups is higher in males than in females [50–53]. In this study, the males had lower *6p21.33* and *AHRR* methylation levels than the females, whereas the decreased methylation of *6p21.33* was associated with CHD according to our results, and the hypomethylation of *6p21.33* and *AHRR* were considered as predictors for the increased cardiovascular mortality [20]. These results suggested that methylation may be one of the molecular mechanisms that lead to gender differences in cardiovascular disease. It seems that the blood-based *6p21.33* and *AHRR* methylations were not influenced by hypertension, diabetes, levels of TC, TG, HDL, and LDL. Given the above, our investigation suggested that the methylation of *6p21.33* and *AHRR* in blood could hardly be influenced by most of the environmental factors, especially the blood lipid index. The signatures of methylation could also be influenced by treatment [54]. Among the 12 cardiovascular drugs available, only 4 drugs showed associations with the methylation level of individual sites on *6p21.33* and *AHRR*. However, these correlations should be taken with caution due to the limited sample size. To further explore the relationship between medication and CHD-related methylation, it is meaningful to conduct the study with an expanded sample size and more adequate information about treatment.

In our study, MALDI-TOF mass spectrometry was used for the quantification of DNA methylation levels. In clinical, mass spectrometry has already been used for multiplex genetic analyses, including non-invasive prenatal tests, disease-related SNP analyses, and even for the detection of Covid-19 [55]. With a semi-quantitative setting, mass spectrometry offers a self-designable, easy-to-use, high throughput, robustness, and cost-saving technique for the candidate-approached DNA methylation analyses. Recent studies have suggested multiple genes are involved in diseases including CHD. With the capacity of supporting multiplex analyses for a panel of genetic and epigenetic variations in a cost-efficient manner, mass spectrometry would have a great potential for clinical utility, as well as for the CHD diagnosis. Our study analyzed the DNA methylation in whole blood or said mainly from the leukocytes. In lack of the possibility to sort the leukocytes from hospital-based samples freshly, we could not further explore the origination of such altered methylation patterns from which cell subtype. However, given that in most clinical and epidemiological settings, it is more readily and convenient to obtain DNA from whole blood, and process it for further clinical practice. Meanwhile, our study was an exploratory investigation on the assessment of CHD risk using blood-based methylation biomarkers based on a limited subject with limited environmental and medication information, our observations need to be validated in multi-center and prospective studies. Collecting abundant lifestyle and historical treatment materials in future studies with large numbers of participants would be appreciated.

## Conclusion

In summary, *6p21.33* methylation, especially at the *6p21.33* cg06126421 site, exhibits a significant association with CHD. This correlation is more susceptible in men and smokers, and more likely appears in CHD patients with heart failure and patients with early cardiac dysfunction. In contrast to Caucasians, the blood DNA methylation at *AHRR* is barely related to the status of CHD in the Chinese population. The combination of *6p21.33* methylation and conventional risk factors might be an intermediate step towards the risk evaluation and detection of CHD.

## Supplementary Information

**Additional file 1**.** Supplementary Figure 1**. Schematic diagrams and the sequences of* 6p21.33* amplicon and* AHRR* amplicon. (A) The location of the investigated 398 bp amplicon in* 6p21.33* and the 5 measurable CpG sites. The* 6p21.33* amplicon covers the main hit CpG site cg06126421 (chr6:30,720,081, build 37/hg19). (B) The location of the investigated 386 bp amplicon in* AHRR* and the 14 measurable CpG sites. The* AHRR* amplicon is located at the third intron of* AHRR*, and covers the main hit CpG site cg05575921 (chr5:373,378, build 37/hg19). (C) The sequence of the* 6p21.33* amplicon examined by the EpiTyper assay (chr6:30,719,778-30,720,175, build 37/hg19, defined by the UCSC Genome Browser). The EpiTyper assay determined the methylation levels of 5 CpGs in this amplicon, and yielded 4 distinguishable mass peaks. The CpG sites that could be measured are in bold, cg06126421 is in bold and underlined. (D) The sequence of the* AHRR* amplicon examined by the EpiTyper assay (chr5:373,077-373,462, build 37/hg19, defined by the UCSC Genome Browser). The EpiTyper assay determined the methylation levels of 14 CpGs in this amplicon, and yielded 10 distinguishable mass peaks. The CpG sites that could be measured are in bold, cg05575921 is in bold and underlined.

**Additional file 2**.** Supplementary Table 1**. Methylation difference of* 6p21.33* and* AHRR* between non-heart failure CHD cases and controls.** Supplementary Table 2**. NYHA classification and the methylation intensity of* 6p21.33* and* AHRR*.

## Data Availability

All data analyzed during the current study are available from the corresponding author upon reasonable request.

## Abbreviations

AHRR: Aryl-hydrocarbon receptor repressor
CHD: Coronary heart disease
OR: Odds ratio
HsCRP: High-sensitivity C-reactive protein
Lp-PLA2: Lipoprotein-associated phospholipase A2
MPO: Myeloperoxidase
BNP: B-type natriuretic peptide
NT-pro BNP: N-terminal prohormone BNP
CpG: Cytidine-phosphate-guanosine
AhR: Aryl hydrocarbon receptor
MI: Myocardial infarction
NYHA: New York Heart Association
TC: Total cholesterol
TG: Triglyceride
HDL: High-density lipoprotein
LDL: Low-density lipoprotein
EDTA: Ethylene diamine tetraacetic acid
MALDI-TOF: Matrix-assisted laser desorption/ionization time-of-flight
IQR: Interquartile range
CIs: Confidence intervals
ROC: Receiver operating characteristic
IER3: Immediate early response 3
MDC1: Mediator of DNA damage checkpoint 1
DHX16: DEAH-box helicase 16
FLOT1: Flotillin 1
TUBB: Tubulin beta class I
PPP1R18: Protein phosphatase 1 regulatory subunit 18
NRM: Nurim
