# Supplementary material for: The association between DNA methylation of 6p21.33 and AHRR in blood and coronary heart disease in Chinese population
Source: BMC Cardiovasc Disord. 2022 Aug 13;22:370. doi: 10.1186/s12872-022-02766-8 (PMC9375073; doi:10.1186/s12872-022-02766-8)
Supplement: Supplementary file 1 — Additional file 1. Supplementary Figure 1. Schematic diagrams and the sequences of 6p21.33 amplicon and AHRR amplicon. (A) The location of the investigated 398 bp amplicon in 6p21.33 and the 5 measurable CpG sites. The 6p21.33 amplicon covers the main hit CpG site cg06126421 (chr6:30,720,081, build 37/hg19). (B) The location of the investigated 386 bp amplicon in AHRR and the 14 measurable CpG sites. The AHRR amplicon is located at the third intron of AHRR, and covers the main hit CpG site cg05575921 (chr5:373,378, build 37/hg19). (C) The sequence of the 6p21.33 amplicon examined by the EpiTyper assay (chr6:30,719,778-30,720,175, build 37/hg19, defined by the UCSC Genome Browser). The EpiTyper assay determined the methylation levels of 5 CpGs in this amplicon, and yielded 4 distinguishable mass peaks. The CpG sites that could be measured are in bold, cg06126421 is in bold and underlined. (D) The sequence of the AHRR amplicon examined by the EpiTyper assay (chr5:373,077-373,462, build 37/hg19, defined by the UCSC Genome Browser). The EpiTyper assay determined the methylation levels of 14 CpGs in this amplicon, and yielded 10 distinguishable mass peaks. The CpG sites that could be measured are in bold, cg05575921 is in bold and underlined. [file 12872_2022_2766_MOESM1_ESM.doc]

**A**

**
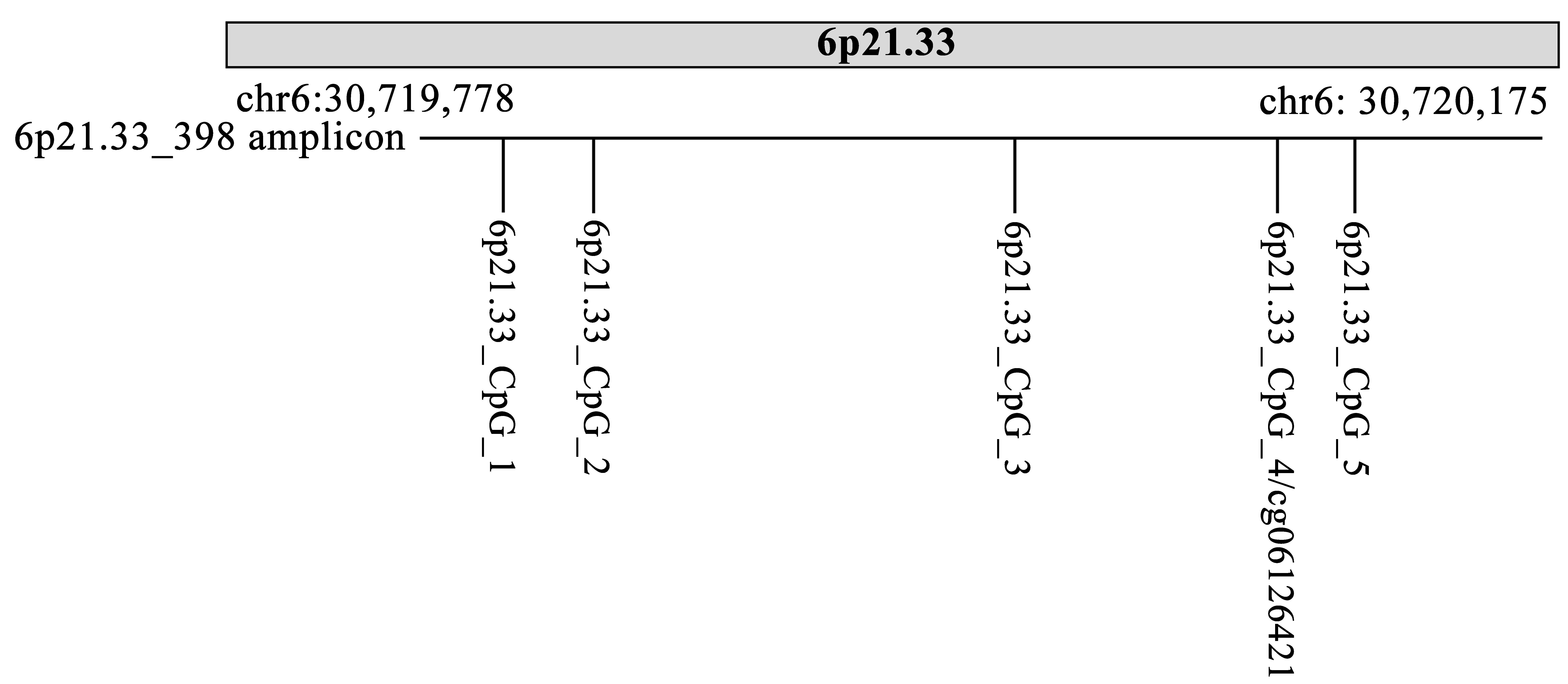
**

**B**

**
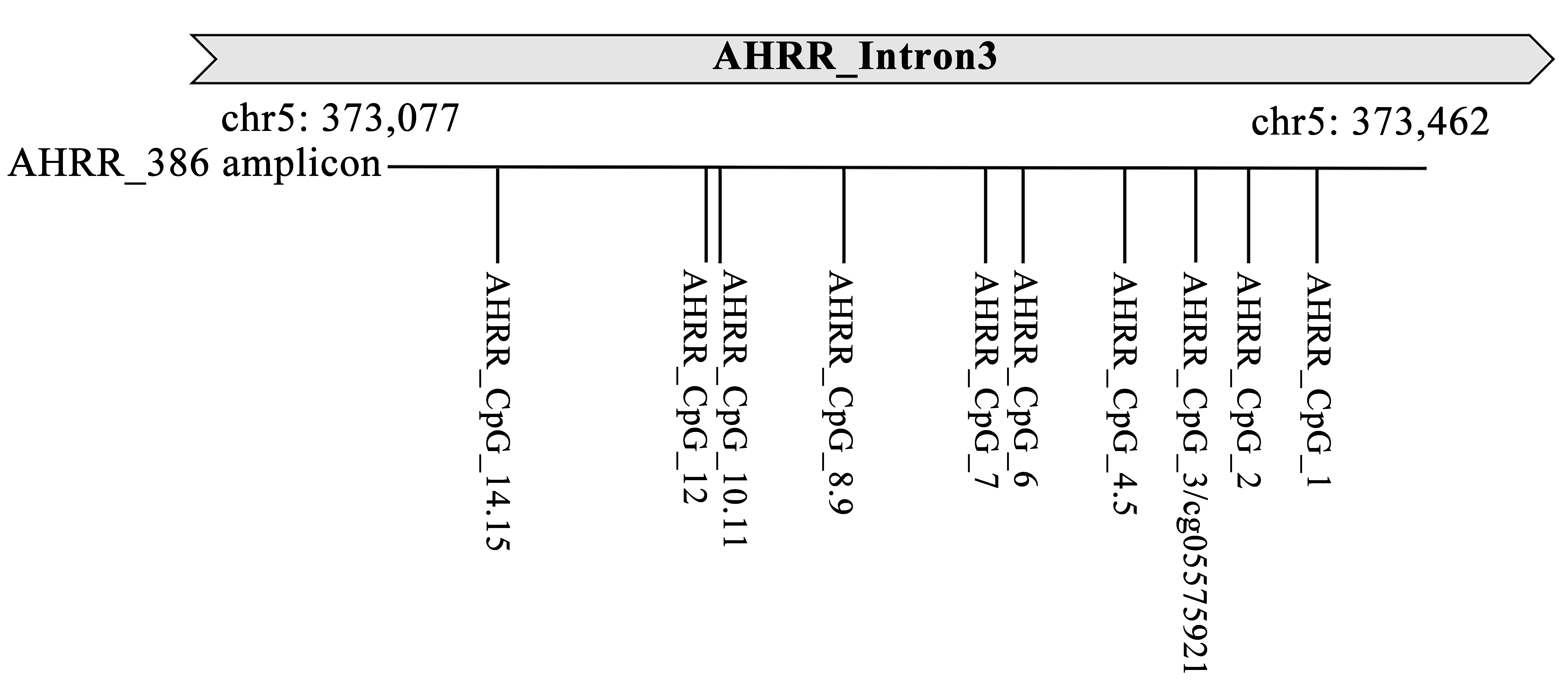
**

**C**

GGCTGCTGAAAAGGCTAGAAATACAGGAT**CG**TTGATGTCATTACTGTTACAGTGCTGTTCC**CG**TGGGAAGCAGCCTGCATCTCTCTGTAGACAGCTCCAATGTCAGCCATTGGAGAATTTGATGGAGATTGAAGTCAAAACATAATCATAGGGGAAAAATGCACAACAAATAAAATATTTCCCCTTTTGTCCAGACTTTTCAGACACCC**CG**TATTTACTGGATGAGTTGATCTACAAAGTGAAAGGCATAAATTAAAGGCTCTCTAATGTTTCTCCTCCTCTGAAACCCCATGATTCAGCAG**CG**TCTCCCATGTGTGCTCAAAAGTTGGA**CG**TGCCTCCAGCCTACCCTCCAAAGCAGTTGCCATAGGCTCTTTTCTTCCAAGGTTGGGAGGGACAGT

**D**

GAGGGGCCCTGCCAGGACCACTCCCAGGGCCCACCCCT**CG**GGCAATGAGAGGCTGGAAGGCTG**CG**TGTCAGATGAGACCCAGC**CG**GGTGCAGGGCAGGGCCAGGGC**CGCG**GGGCAGGGCTGCCCTCTCAGAGGTGAACAGTCCCCA**CG**GCTGGGCCTGAACC**CG**GATCTGTAGCCTGGGCAACCCCTGTGTCCTGCCAGCACTCTGCCAAC**CGCG**CAGAACCTCAGCCACCTTGGAGAGTCTCAGGGCCATTACCAGC**CG**TC**CG**CCCA**CG**GCTGCAGAGTTCACTGCAACCAAGAAGGGGCTCATTTGGAACAGCGAGAATGTGAAGTCAGCACCAAGGCCT**CG**GG**CG**ACCCTGTGCCCACAGCAAGGGCTGGAGCAGAGTGGCTC

**Supplementary Figure 1.** Schematic diagrams and the sequences of *6p21.33* amplicon and *AHRR* amplicon. **(A)** The location of the investigated 398 bp amplicon in *6p21.33* and the 5 measurable CpG sites. The *6p21.33* amplicon covers the main hit CpG site cg06126421 (chr6:30,720,081, build 37/hg19). **(B)** The location of the investigated 386 bp amplicon in *AHRR* and the 14 measurable CpG sites. The *AHRR* amplicon is located at the third intron of *AHRR*, and covers the main hit CpG site cg05575921 (chr5:373,378, build 37/hg19). **(C)** The sequence of the *6p21.33* amplicon examined by the EpiTyper assay (chr6:30,719,778-30,720,175, build 37/hg19, defined by the UCSC Genome Browser). The EpiTyper assay determined the methylation levels of 5 CpGs in this amplicon, and yielded 4 distinguishable mass peaks. The CpG sites that could be measured are in bold, cg06126421 is in bold and underlined. **(D)** The sequence of the *AHRR* amplicon examined by the EpiTyper assay (chr5:373,077-373,462, build 37/hg19, defined by the UCSC Genome Browser). The EpiTyper assay determined the methylation levels of 14 CpGs in this amplicon, and yielded 10 distinguishable mass peaks. The CpG sites that could be measured are in bold, cg05575921 is in bold and underlined.


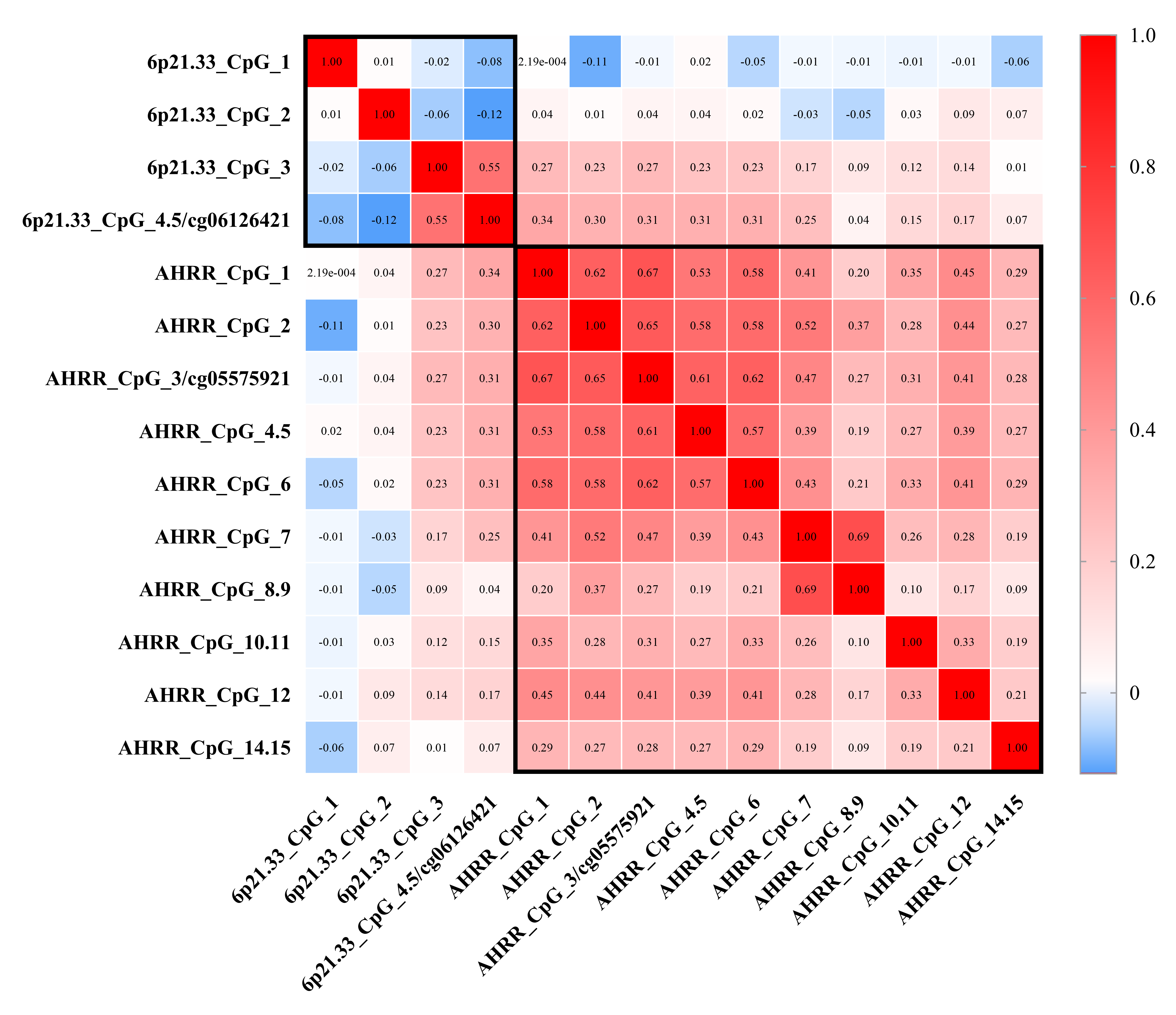


**Supplementary Figure 2**. A heatmap of correlations among the CpG sites in the *6p21.33* amplicon and *AHRR* amplicon. The positively correlated CpG sites are in red, and the negatively correlated CpG sites are in blue. The color bar represents the strength of correlation as Spearman Rho. The CpG sites in the *6p21.33* amplicon and *AHRR* amplicon are framed respectively.

**
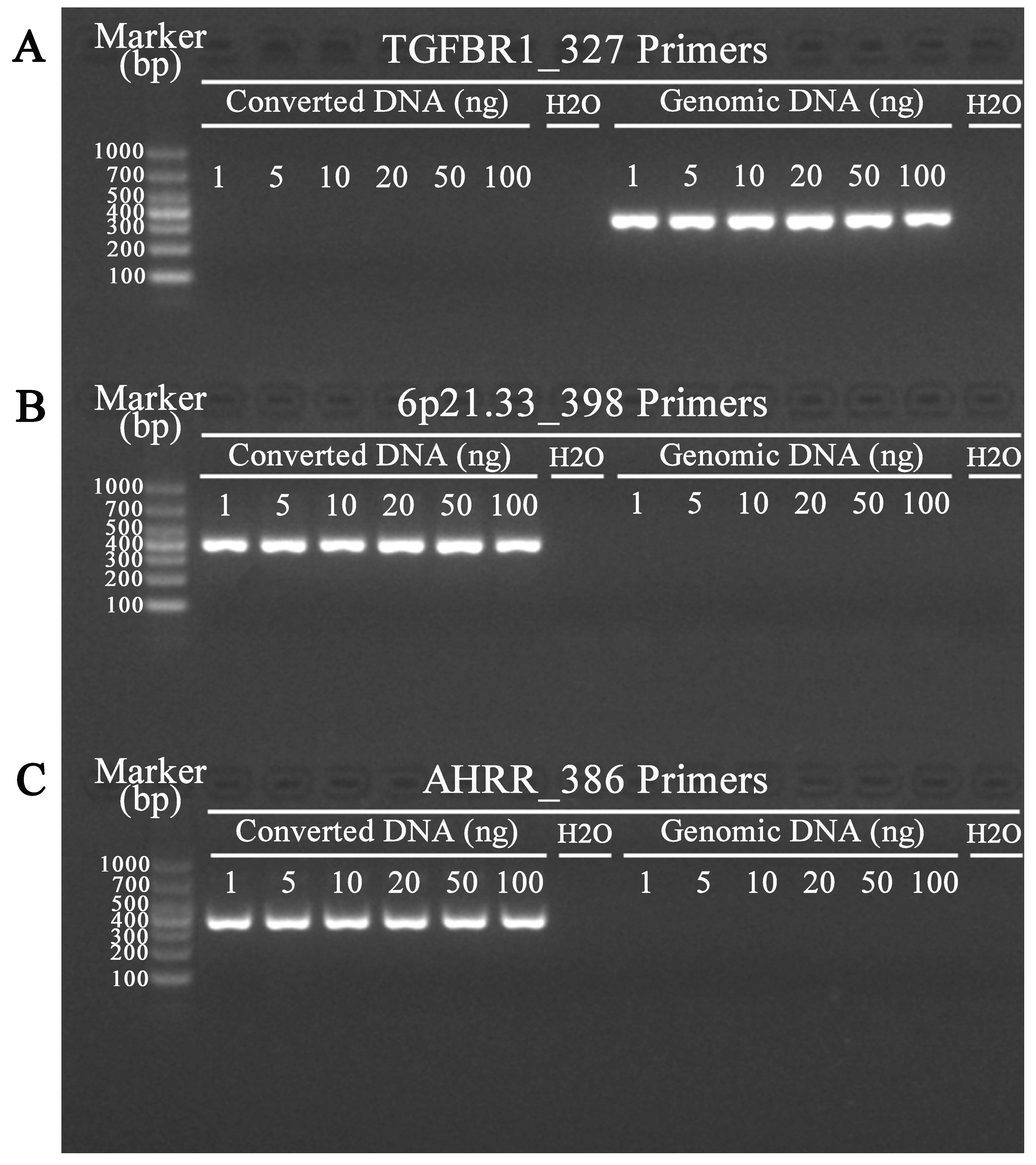
**

**Supplementary Figure 3**. Assessment of the bisulfite conversion efficiency. Variant amount of bisulfite converted DNA and original genomic DNA (1, 5, 10, 20, 50, and 100 ng, respectively) was used as the template for the PCR amplification of different pairs of primers. Water was used as a negative control. **(A)** 327 bp PCR products amplified by the *TGFBR1* primers (primers specific for the original genomic DNA) **(B)** 398 bp PCR products amplified by the *6p21.33* methylation primers (primers specific for the bisulfite converted DNA) **(C)** 386 bp PCR products amplified by the *AHRR* methylation primers (primers specific for the bisulfite converted DNA).
