# Supplementary material for: The association between DNA methylation of 6p21.33 and AHRR in blood and coronary heart disease in Chinese population
Source: BMC Cardiovasc Disord. 2022 Aug 13;22:370. doi: 10.1186/s12872-022-02766-8 (PMC9375073; doi:10.1186/s12872-022-02766-8)
Supplement: Supplementary file 2 — Additional file 2. Supplementary Table 1. Methylation difference of 6p21.33 and AHRR between non-heart failure CHD cases and controls. Supplementary Table 2. NYHA classification and the methylation intensity of 6p21.33 and AHRR. [file 12872_2022_2766_MOESM2_ESM.doc]

**The association between DNA methylation of *6p21.33* and *AHRR* in blood and coronary heart disease in Chinese population**

**SUPPLEMENTARY TABLES**

**Supplementary Table 1. Methylation difference of *6p21.33* and *AHRR* between non-heart failure CHD cases and controls**

| **CpG sites** | **Controls (N=184)** | **Non-heart failure CHD cases (N=35)** | ***p*-value** |
| --- | --- | --- | --- |
|  | **Median (IQR)** | **Median (IQR)** |  |
| 6p21.33_CpG_1 | 1.00(0.99-1.00) | 1.00(0.95-1.00) | 0.066 |
| 6p21.33_CpG_2 | 0.93(0.90-0.95) | 0.94(0.91-0.98) | 0.082 |
| 6p21.33_CpG_3 | 0.63(0.57-0.70) | 0.65(0.58-0.72) | 0.451 |
| 6p21.33_CpG_4.5/cg06126421 | 0.50(0.45-0.55) | 0.49(0.42-0.55) | 0.492 |
| AHRR_CpG_1 | 0.73(0.63-0.80) | 0.73(0.61-0.89) | 0.328 |
| AHRR_CpG_2 | 0.87(0.74-0.96) | 0.92(0.74-1.00) | 0.173 |
| AHRR_CpG_3/cg05575921 | 0.77(0.64-0.83) | 0.76(0.54-0.86) | 0.679 |
| AHRR_CpG_4.5 | 0.77(0.68-0.84) | 0.76(0.65-0.88) | 0.859 |
| AHRR_CpG_6 | 0.84(0.74-0.90) | 0.84(0.64-0.96) | 0.689 |
| AHRR_CpG_7 | 0.66(0.53-0.76) | 0.61(0.48-0.71) | 0.087 |
| AHRR_CpG_8.9 | 0.85(0.73-0.94) | 0.79(0.75-0.88) | 0.083 |
| AHRR_CpG_10.11 | 0.92(0.89-0.95) | 0.93(0.85-0.97) | 0.606 |
| AHRR_CpG_12 | 0.88(0.81-0.95) | 0.88(0.76-1.00) | 0.644 |
| AHRR_CpG_14.15 | 0.94(0.91-0.95) | 0.93(0.87-0.97) | 0.533 |

^a^The *p*-values were calculated by the Mann-Whitney test, and significant *p*-values are in bold. *AHRR* aryl-hydrocarbon receptor repressor, *CHD* coronary heart disease, *CpG* cytidine-phosphate-guanosine, *IQR* interquartile range.

**Supplementary Table 2. NYHA classification and the methylation intensity of *6p21.33* and *AHRR***

| **CpG sites** | **NYHA Ⅰ&Ⅱ CHD cases (N=124)** | **NYHA Ⅲ&Ⅳ CHD cases (N=37)** | ***p*-value** |
| --- | --- | --- | --- |
|  | **Median (IQR)** | **Median (IQR)** |  |
| 6p21.33_CpG_1 | 1.00(1.00-1.00) | 1.00(0.98-1.00) | 0.301 |
| 6p21.33_CpG_2 | 0.94(0.91-0.97) | 0.93(0.89-0.95) | 0.163 |
| 6p21.33_CpG_3 | 0.62(0.53-0.69) | 0.59(0.52-0.70) | 0.344 |
| 6p21.33_CpG_4.5/cg06126421 | 0.49(0.44-0.52) | 0.44(0.39-0.54) | 0.330 |
| AHRR_CpG_1 | 0.74(0.59-0.82) | 0.72(0.51-0.83) | 0.782 |
| AHRR_CpG_2 | 0.89(0.72-1.00) | 0.86(0.65-0.99) | 0.252 |
| AHRR_CpG_3/cg05575921 | 0.75(0.60-0.85) | 0.77(0.56-0.85) | 0.927 |
| AHRR_CpG_4.5 | 0.76(0.61-0.84) | 0.70(0.57-0.81) | 0.223 |
| AHRR_CpG_6 | 0.83(0.68-0.90) | 0.76(0.64-0.91) | 0.382 |
| AHRR_CpG_7 | 0.69(0.55-0.82) | 0.66(0.49-0.79) | 0.214 |
| AHRR_CpG_8.9 | 0.89(0.78-0.94) | 0.85(0.74-0.94) | 0.230 |
| AHRR_CpG_10.11 | 0.92(0.88-0.95) | 0.92(0.89-0.96) | 0.948 |
| AHRR_CpG_12 | 0.89(0.80-0.95) | 0.91(0.81-1.00) | 0.320 |
| AHRR_CpG_14.15 | 0.94(0.91-0.96) | 0.96(0.89-0.99) | 0.115 |

^a^The *p*-values were calculated by the Mann-Whitney test, and significant *p*-values are in bold. *NYHA* New York Heart Association.
